# Supplementation with Complex Dietary Fiber during Late Pregnancy and Lactation Can Improve Progeny Growth Performance by Regulating Maternal Antioxidant Status and Milk Quality

**DOI:** 10.3390/antiox13010022

**Published:** 2023-12-21

**Authors:** Xinyu Liu, Xinke Wei, Ye Feng, Huawei Liu, Jiaqi Tang, Feng Gao, Baoming Shi

**Affiliations:** College of Animal Science and Technology, Northeast Agricultural University, Harbin 150030, China; s220502026@neau.edu.cn (X.L.); s220501901@neau.edu.cn (X.W.); s220501029@neau.edu.cn (Y.F.); s210501032@neau.edu.cn (H.L.); s220502024@neau.edu.cn (J.T.); b210501006@neau.edu.cn (F.G.)

**Keywords:** complex dietary fiber, sow, antioxidant, piglet, growth performance

## Abstract

This study investigated the nutritional benefits of complex dietary fiber (beta-glucan and fructo-oligosaccharides, CDF) supplementation in sows and piglets during late pregnancy and lactation. Twenty-four sows were randomly divided into two groups: the control group was fed a basal diet (*n* = 12), and the experimental group was fed a CDF diet (0.25% CDF replaced the same proportion of corn in the basal diet, *n* = 12). Dietary treatment was given from day 107 of pregnancy to day 25 of lactation. The results of this experiment showed that CDF increased the average daily feed intake (ADFI) of sows during lactation and the weaning body weight (BW) and average daily gain of piglets. Dietary CDF supplementation improved the antioxidant capacity and immune level of sows and decreased the serum zonulin level. Dietary supplementation with CDF increased the levels of antioxidant activity, immunoglobulin, and anti-inflammatory factor interleukin-10 (IL-10) in milk. Meanwhile, piglets in the CDF group had increased serum antioxidant activity, immunoglobulin, and growth-related hormone levels; decreased malondialdehyde (MDA), interleukin-6 (IL-6), and D-lactic acid (D-LA) levels; and increased fecal short-chain fatty acid content. In addition, the CDF group increased the diversity of microorganisms in sow feces. In conclusion, the supplementation of a diet with CDF in late pregnancy and lactation can alleviate the oxidative stress of sows, improve milk quality, and have significant positive effects on the antioxidant capacity and growth performance of piglets.

## 1. Introduction

The intense catabolic state of sows in late pregnancy and lactation can lead to elevated levels of oxidative stress, which not only negatively regulates the reproductive performance and lactation performance of sows but also negatively affects the health and growth of offspring [1,2]. Specifically, oxidative stress leads to reduced feed intake in sows during lactation, resulting in long-term negative energy balance, increased loss of physical condition, and decreased milk production and quality [3]. Newborn piglets also experience a high risk of oxidative stress and are very vulnerable to free radical oxidative damage, most commonly causing oxidative stress and inflammatory responses in the gut [4,5]. In addition, the immunity of newborn piglets mainly comes from a variety of immunoglobulins and cytokines in the sow’s colostrum and milk, which can stimulate the maturation of the immune system [6]. Therefore, the dynamic balance between antioxidant systems and free radicals in late gestation and lactating sows is critical for sows and offspring [7]. So far, the implementation of effective nutrition strategies in late pregnancy and lactation may be an effective way to alleviate oxidative stress in sows and improve the immunity and growth and development of offspring [3].

Dietary fiber is an edible carbohydrate polymer that is not digested in the small intestine and is fermented by microorganisms in the large intestine to produce short-chain fatty acids (SCFAs), thereby positively affecting the host’s intestinal health, immune regulation, and metabolic function [8,9]. It has been demonstrated to positively influence the diversity of intestinal microflora, the integrity of barrier function, and immunity in weaned and fattening pigs [10,11,12]. In addition, plant dietary fiber is considered a natural, innocuous, and residual-free feed additive that can be used as a pathway to replace antibiotics [3]. Beta-glucan is a rich polysaccharide that is widely distributed in nature and is found In bacteria, fungi, plants, and algae [13]. Fructo-oligosaccharides are low-grade polymeric sugars, which are widely found in bananas, barley, garlic, and other plants. Furthermore, both beta-glucan and fructo-oligosaccharides have dietary fiber properties such as regulating antioxidant capacity, activating the immune system, and improving gastrointestinal health [10,14].

Functional dietary fiber inulin, chitosan oligosaccharides, and konjac glucomannan have been shown to effectively regulate oxidative stress in sows during late pregnancy and lactation and improve the growth of offspring [13,15,16]. However, there is limited research on the effects of dietary mixtures of beta-glucan and oligosaccharides on oxidative stress in sows and the growth health of offspring during late pregnancy and lactation. Therefore, the present study aimed to investigate the effects of CDF addition during late gestation and lactation on the performance, antioxidant capacity, immune levels, intestinal permeability, and short-chain fatty acids of sows and piglets. Furthermore, colostrum and milk composition, antioxidant levels, immunoglobulins, and cytokines were evaluated to explore the underlying mechanisms.

## 2. Material and Methods

### 2.1. Animals, Diets, and Management

The Northeast Agricultural University Ethical Committee for Animal Experiments (Grant Number: NEAU-2013-9) approved all animal experiments conducted at the Bayan Kangrun Husbandry Co. (Inner Heilongjiang, China). The experiment was a stratified randomized group design: twenty-four Large White × Landrace sows (mean backfat thickness, 18.47 ± 0.72 mm, parity, 3–6) that gave birth during the same period were selected and subsequently stratified according to BW, backfat thickness, and parity and randomly divided into the control (*n* = 12) and the experimental (*n* = 12) groups in a 1:1 ratio. The control group (CON) was fed a basal diet, and the complex dietary fiber group (CDF) was supplemented with a 0.25% mixture of beta-glucan and fructo-oligosaccharides (1:1) to replace the same proportion of corn in the basal diet. There was no difference in the energy content of the diets of the CDF group and the basal diet. The sow basal diet in Table S1 was formulated to meet or exceed the nutritional requirements of gestating and lactating sows according to the guidelines provided by the National Research Council (NRC, 2012). The feeding experiment started from d 107 of gestation to d 25 of lactation (weaning).

On d 107 of gestation, sows were transferred to the farrowing house and housed in separate 2.1 × 1.5 m farrowing pens. The sows were fed 3 to 3.5 kg/d diets from d 107 to 114 of gestation. On the day of farrowing, 0.5 kg of diet was fed, and then the amount of feed was gradually increased by 1 kg per day until the seventh day of lactation; afterwards, the sows were given free choice of diet until weaning. The ADFI of the sows was recorded throughout the lactation period. All sows and piglets had free access to water during the experiment. Litter size was adjusted to 14 to 15 piglets by crossfading within 24 h after farrowing. The average temperature of the birthing house was maintained at 26–28 °C. In this study, breast milk was the only source of nutrition for piglets.

### 2.2. Sample and Data Collection

Sow backfat thickness was determined on day 107 of gestation and day 25 of lactation. After parturition, we recorded the number of piglets born alive, newborn weight, and litter weight for each sow. The number of weaning piglets, body weight (BW), and litter weight were recorded on d 25 of lactation. Six sows (similar parity and backfat thickness) were selected for each group, colostrum was collected within 2 h after delivery of the first piglet, and milk was collected on the 25th day of lactation and stored at −80 °C for analysis. In each group, 6 sows (the same sows as the milk-collecting sows) and 6 piglets (from the 6 milk-collecting sows, 1 piglet from each litter close to the average weight of the litter) were selected, and their fresh feces were collected on the 25th day of lactation and stored at −80 °C to be analyzed. Venous blood samples were collected from 6 sows (same sows as those selected for fecal sampling) and 6 piglets (same piglets as those selected for fecal sampling) from each group on the 25th day of lactation, centrifuged at 3000 rpm for 10 min to obtain serum, and stored at −80 °C for analysis.

### 2.3. Milk Composition

Fat, protein, lactose, and total solids in colostrum and milk samples were analyzed using a fully automated milk composition analyzer (Milko Scan TM FT + Analyzer, Foss, Hilleroed, Denmark).

### 2.4. Determination of Antioxidant Capacity in Serum and Milk of Sows and Piglets

The activities of superoxide dismutase (SOD) and glutathione peroxide (GPH-Px), the total antioxidant capacity (T-AOC), and the level of malondialdehyde (MDA) were determined usingassay kits (Nanjing Jiancheng Biology Engineering Research Institute, Nanjing, China). The experimental procedure was strictly carried out according to the instructions.

### 2.5. Measurement of Immunity, Inflammation, and Intestinal Permeability Indices in Sows and Piglets

The levels of immunoglobulin A (IgA), immunoglobulin G (IgG), immunoglobulin M (IgM), interleukin-6 (IL-6), interleukin-10 (IL-10), tumor necrosis factor-alpha (TNF-α), diamine oxidase (DAO), D-lactic acid (D-LA), endotoxin (ET), and zonulin were measured using Enzyme-Linked Immunosorbent Assay (ELISA) kits from Shanghai Enzyme-linked Biotechnology Co., Ltd. (Shanghai, China). The experimental procedures were performed in strict accordance with the manufacturer’s instructions.

### 2.6. Measurement of Immune and Inflammatory Indices in Colostrum and Milk

The ELISA kits (Shanghai Enzyme-linked Biotechnology Co., Ltd., Shanghai, China) determined the activities of IgA, IgM, IgG, IL-6, IL-10, and TNF-α in colostrum and milk. The experimental procedures were carried out in strict accordance with the instructions.

### 2.7. Piglet Serum Reproduction and Sow Serum Feeding-Related Hormones

The concentrations of growth hormone (GH), insulin-like growth factor (IGF), peptide YY (PYY), and glucagon-like peptide-1 (GLP-1) were determined using ELISA kits (Shanghai Enzyme-linked Biotechnology Co., Ltd., Shanghai, China) according to the manufacturer’s instructions.

### 2.8. Fecal Short-Chain Fatty Acids and Water Content Analysis

SCFAs in feces sample were quantitatively analyzed via gas chromatography. Specifically, 2 g samples were dissolved in 2 mL ultra-pure water, leached at 4 °C for 48 h, and then centrifuged at 4 °C at 10,000 rpm/min for 10 min and repeated twice, after which the supernatant was taken and filtered by the filter membrane. Then, the internal standard (25% metaphosphate solution containing crotonic acid) was mixed with the supernatant at the volume ratio of 1:5. Finally, the mixture was centrifuged at 10,000 rpm/min for 10 min, and the supernatant was extracted for GC system analysis. The fecal samples of sows were dried at 70 °C for 72 h, and then the fecal water content was calculated using the equation: water content of feces = (wet weight of feces − dry weight of feces)/wet weight of feces × 100%.

### 2.9. DNA Extraction, PCR, and Library Construction and Sequencing

Total genomic DNA was extracted from the sow feces sample using the CTAB/SDS method. DNA concentration and purity were monitored on 1% agarose gel. Primes 341F(5′-CCTAYGGGRBGCASCAG-3′) and 805R(5′-GGACTACNNGGGTATCTAAT-3′) were used to construct PCR amplification for the V3-V4 region of bacterial 16S rDNA gene. The amplified products were purified using a German Zeigen gel extraction kit. The sequencing library was then generated using the NEBNext^®^Ultra™IIDNA Library Preparation Kit. The library quality was evaluated using the Qubit@2.0 fluorescence analyzer (Thermo Science, Waltham, MA, USA) and the Agilent Bioanalyzer 2100, Santa Clara, CA, USA. Finally, the libraries were sequenced on the Illumina NovaSeq platform to generate 250 bp paired-end reads.

### 2.10. Statistical Analysis

First, Microsoft Excel 2020 was used to sort out the experimental data; then, SPSS (version 23; IBM Corporation, Armonk, NY, USA) was used to conduct independent sample *t*-test, and the analysis index was taken as the unit of repetition. The experimental data were expressed as mean ± SEM, with statistical significance denoted as * *p* < 0.05 and ** *p* < 0.01 *** *p* < 0.001. The correlation analysis was performed by using Pearson correlation.

## 3. Results

### 3.1. Animal Performance

The effect of CDF on sow and piglet performance is summarized in Table 1. Compared with the CON group, dietary supplementation with CDF improved (*p* < 0.05) the ADFI of sows and the weaning BW and average daily gain of piglets. There were no significant differences (*p* > 0.05) in the backfat thickness of sows and the litter weight, litter size, and cross-fostering BW of piglets between the two treatment groups.

### 3.2. Fecal Water Content and Serum Feeding-Related Hormones

**Figure 1:**
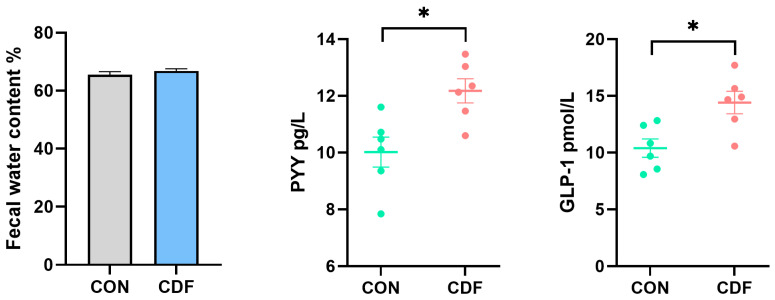
Effects of CDF on fecal water content and feeding-related hormone levels of sows. Data are expressed as mean ± SEM (*n* = 6). * *p* < 0.05. CON: control; CDF: complex dietary fiber.

Sow fecal water content and feeding-related hormones are shown in Figure 1. The supplementation of CDF in late pregnancy and lactation had no significant effect (*p* > 0.05) on the fecal water content of the sows, but it significantly increased (*p* < 0.05) serum PYY and GLP-1 levels after 4 h of feeding.

### 3.3. Immunization, Intestinal Permeability, and Inflammation in Sows

**Figure 2:**
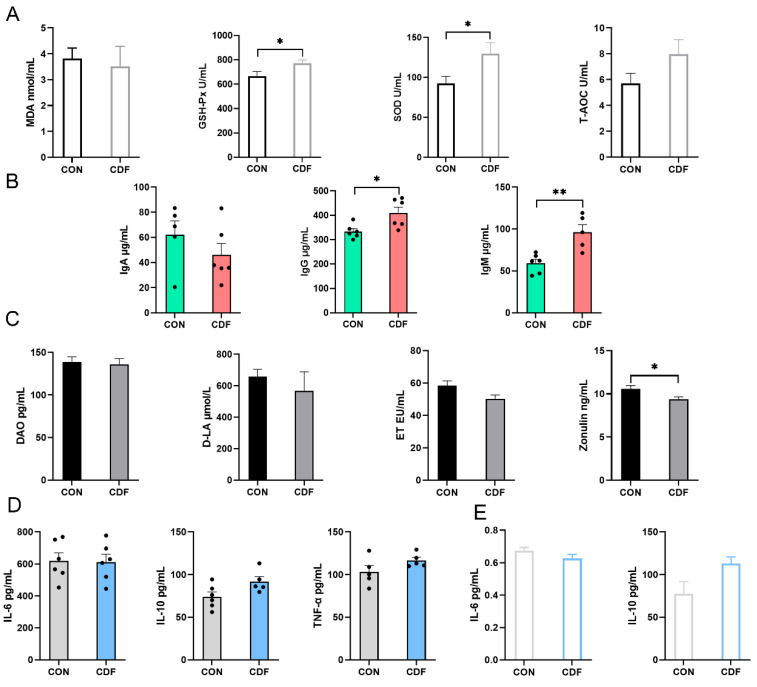
Effects of CDF on serum antioxidant capacity (**A**), immune level (**B**), intestinal permeability (**C**), inflammatory factors (**D**), and fecal inflammatory factors (**E**) of sows. Data are expressed as mean ± SEM (*n* = 6). * *p* < 0.05 ** *p* < 0.01. CON: control; CDF: complex dietary fiber.

As shown in Figure 2A, the dietary treatment had no effect (*p* > 0.05) on serum MDA and T-AOC, but it significantly increased (*p* < 0.05) serum GSH-Px and SOD. There was no significant change (*p* > 0.05) in serum IgA, but IgG (*p* < 0.05) and IgM (*p* < 0.01) were significantly increased after the dietary treatment (Figure 2B). The maternal supplementation of CDF had no significant effect (*p* > 0.05) on DAO, D-LA, and ET in serum, but it significantly reduced (*p* < 0.05) the content of zonulin (Figure 2C). There were no significant differences (*p* > 0.05) in serum IL-6, IL-10, and TNF-α between the two treatment groups (Figure 2D). In addition, IL-6 and IL-10 in feces were not affected (*p* > 0.05) by dietary treatment (Figure 2E).

### 3.4. Composition, Immunity, and Inflammation of Colostrum and Milk

**Figure 3:**
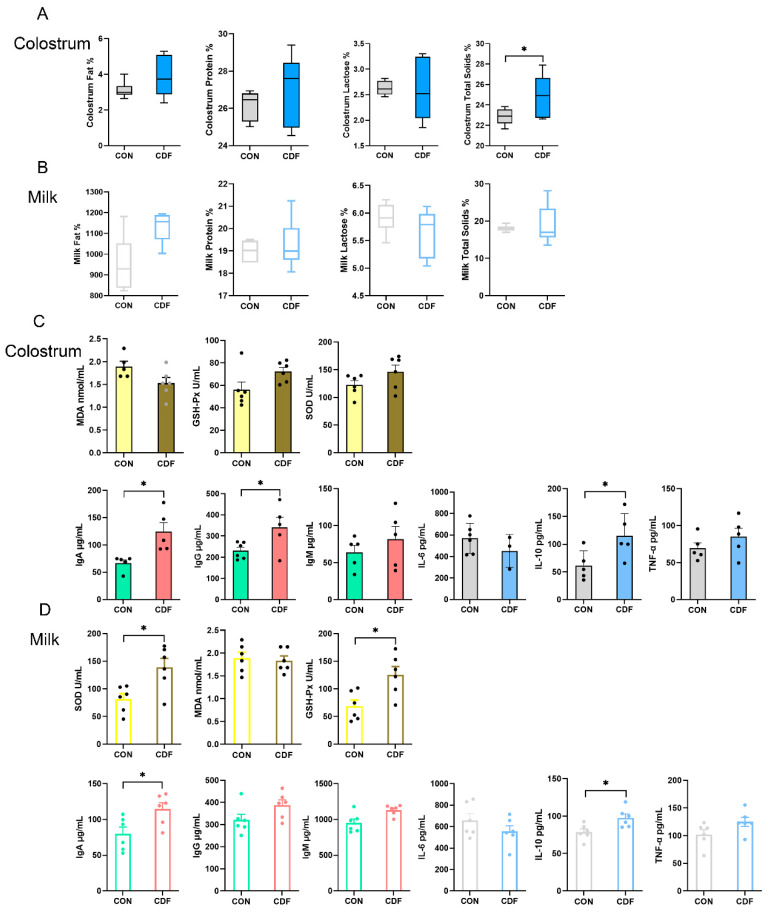
Effect of CDF on colostrum and milk composition (**A**,**B**), antioxidant capacity, immune level, and inflammatory factors for colostrum and milk (**C**,**D**) in sows. Data are expressed as mean ± SEM (*n* = 6). * *p* < 0.05. CON: control; CDF: complex dietary fiber.

As illustrated in Figure 3A, the diet treatment had no significant effect (*p* > 0.05) on the ratio of fat, protein, and lactose in colostrum, but it significantly increased (*p* < 0.05) the total solids. There was no significant difference (*p* > 0.05) in milk composition between the two treatment groups (Figure 3B). The maternal supplementation of CDF significantly increased (*p* < 0.05) the levels of IgA, IgG, and IL-10 in colostrum, and for MDA, GSH-Px, SOD, IgM, IL-6, and TNF-α, there were no differences (*p* > 0.05) between both treatments (Figure 3C). In addition, CDF supplementation significantly increased (*p* < 0.05) the content of SOD, GSH-Px, IgA, and IL-10 in milk, and MDA, IgG, IgM, IL-6, and TNF-α were not significantly different (*p* > 0.05) between the two groups (Figure 3D).

### 3.5. Immunity, Inflammation, Reproduction, Intestinal Permeability in Piglets

**Figure 4:**
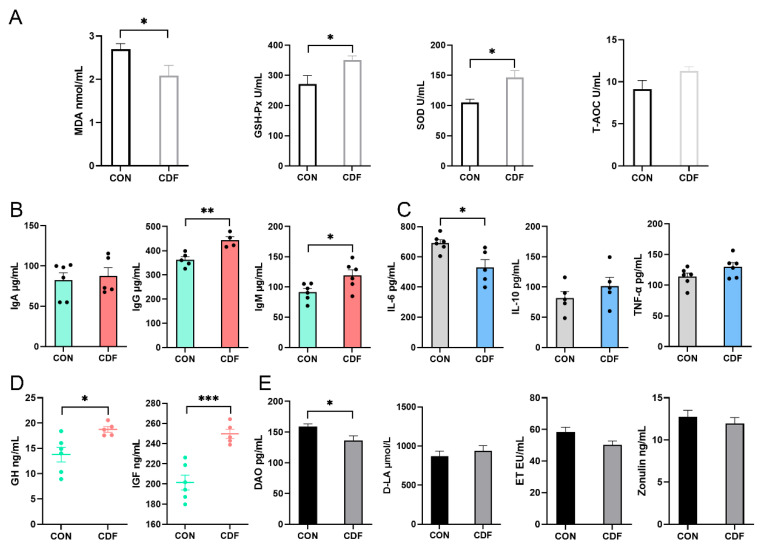
Effects of CDF on serum antioxidant capacity (**A**), immune level (**B**), inflammatory factors (**C**), reproductive hormones (**D**) and intestinal permeability (**E**) of piglets. Data are expressed as mean ± SEM (*n* = 6). * *p* < 0.05 ** *p* < 0.01 *** *p* < 0.001. CON: control; CDF: complex dietary fiber.

As presented in Figure 4A, the activities of GSH-Px and SOD in the serum of the CDF group were significantly increased (*p* < 0.05), and the content of MDA was significantly decreased (*p* < 0.05), but T-AOC was not changed by dietary treatment (*p* > 0.05). The maternal supplementation of CDF significantly increased the levels of IgG (*p* < 0.01) and IgM (*p* < 0.05) in serum, and IgA did not change (*p* > 0.05) significantly (Figure 4B). The levels of serum IL-10 and TNF-α in the CDF group were not significantly changed (*p* > 0.05), but IL-6 was significantly decreased (*p* < 0.05) (Figure 4C). Dietary treatment significantly increased the levels of serum GH (*p* < 0.05) and IGF (*p* < 0.001) (Figure 4D). In addition, maternal supplementation with CDF significantly reduced (*p* < 0.05) the content of serum DAO, but D-LA, ET, and zonulin were similar (*p* > 0.05) in both treatment groups (Figure 4E).

### 3.6. SCFA of Sows and Piglets

**Figure 5:**
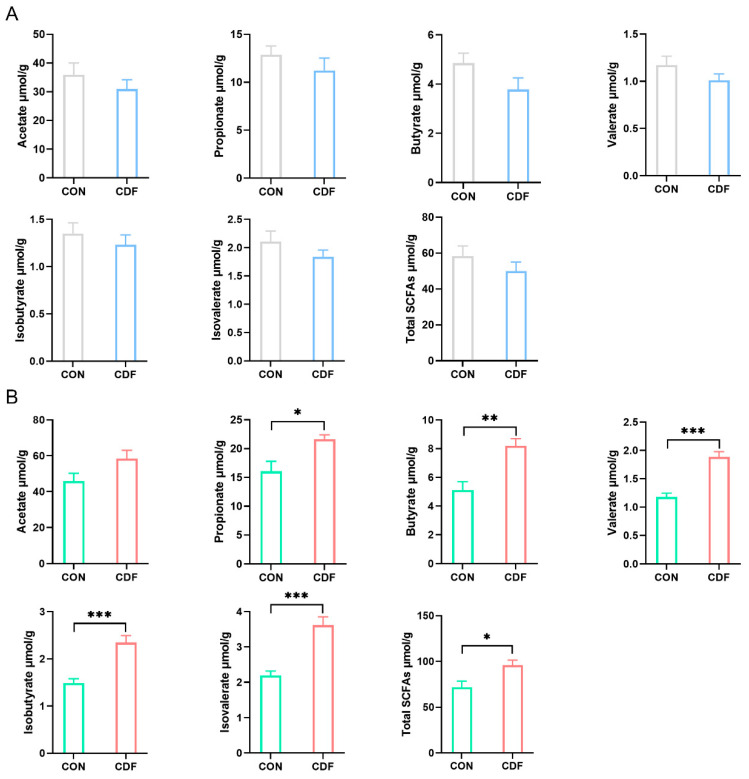
Effects of CDF on SCFAs in sows (**A**) and piglets (**B**). Data are expressed as mean ± SEM (*n* = 6). * *p* < 0.05 ** *p* < 0.01 *** *p* < 0.001. CON: control; CDF: complex dietary fiber.

The effect of CDF on the content of fecal SCFAs in sows and piglets is shown in Figure 5. Maternal dietary fiber supplementation had no remarkable effect (*p* > 0.05) on the proportion of fecal SCFA content in sows (Figure 5A). However, the contents of propionate (*p* < 0.05), butyrate (*p* < 0.01), valerate (*p* < 0.001), isobutyrate (*p* < 0.001), isovalerate (*p* < 0.001), and total SCFAs (*p* < 0.05) in piglet feces were significantly increased by CDF supplementation, and acetate did not significant change (*p* > 0.05) (Figure 5B).

### 3.7. Fecal Microbiota of Sows

**Figure 6:**
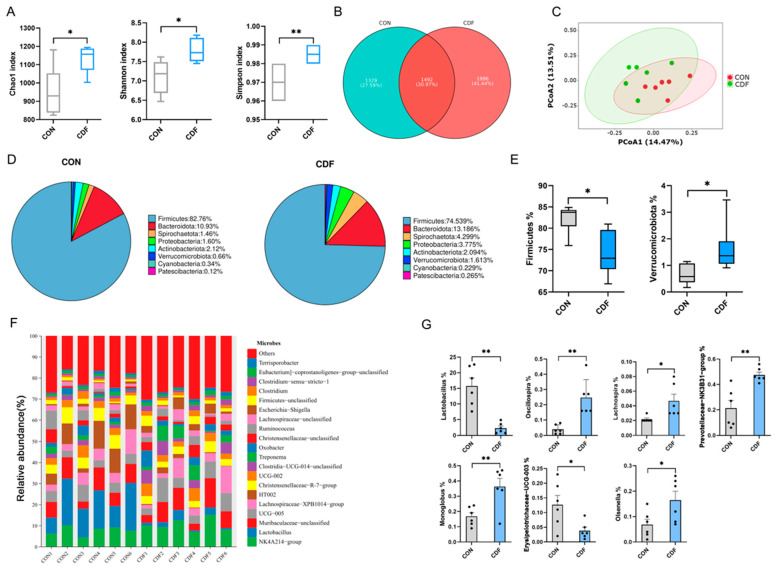
Effect of KGM on the fecal microbiota composition of sows. Comparison of the observed Chao1, Shannon, and Simpson indices (**A**). Venn diagram of shared and specific ASVs in the fecal microbiota between CON and CDF group (**B**). Principal coordinate map based on the unweighted UniFrac index was used to analyze the degree of difference in the fecal microbiota (**C**). Relative abundance of fecal microbiota at the phylum (**D**,**E**) and genus (**F**,**G**) level. Data are expressed as mean ± SEM (*n* = 6). * *p* < 0.05 ** *p* < 0.01. CON: control; CDF: complex dietary fiber.

The effects of CDF addition on the fecal microbiota of sows are shown in Figure 6. The bacterial α-diversity as shown by the Chao1, Shannon, and Simpson indexes was significantly increased in the CDF group (Figure 6A). By observing the Venn diagram, it was found that there were 1492 shared ASVs in both groups and 1392 and 1996 specific ASVs in the CON and CDF groups, respectively (Figure 6B). The unweighted principal coordinate analysis (PCOA) of β-diversity showed significant microbial community separation between both groups, suggesting changes in the gut microbial community caused by maternal CDF supplementation (Figure 6C). At the phylum level, *Firmicutes* and *Bacteroidetes* were the dominant phyla, accounting for more than 85% of the totality (Figure 6D). At the genus level, the dominant genera were *NK4A214_group*, *Lactobacillus*, *Muribaculaceae_unclassified*, *UCG-005*, *Lachnospiraceae_XPB1014_group*, *HT002*, *Christensenellaceae_R-7_group*, *UCG-002*, *Clostridia_UCG-014_unclassified*, *Treponema*, *Oxobacter*, *Christensenellaceae_unclassified,* and *Ruminococcus* (Figure 6F). Subsequently, the difference in microbial composition between the two treatments was further analyzed. At the phylum level, the abundance of *Firmicutes* was significantly reduced (*p* < 0.05) and the abundance of *Verrucomicrobiota* was significantly increased (*p* < 0.05) compared with the CON group (Figure 6E). At the genus level, maternal supplementation with CDF significantly increased the abundance of *Oscillospira* (*p* < 0.01), *Lachnospria* (*p* < 0.05), *Prevotellaceae_NK3B31_group* (*p* < 0.01), Monoglobus (*p* < 0.01), and Olsenella (*p* < 0.05). Meanwhile, the abundance of *lactobacillus* (*p* < 0.01) and *Erysipelotrichaceae_UCG-003* (*p* < 0.05) was significantly reduced (Figure 6G).

### 3.8. Correlation between the Immune Level of Sows and Piglet Growth

**Figure 7:**
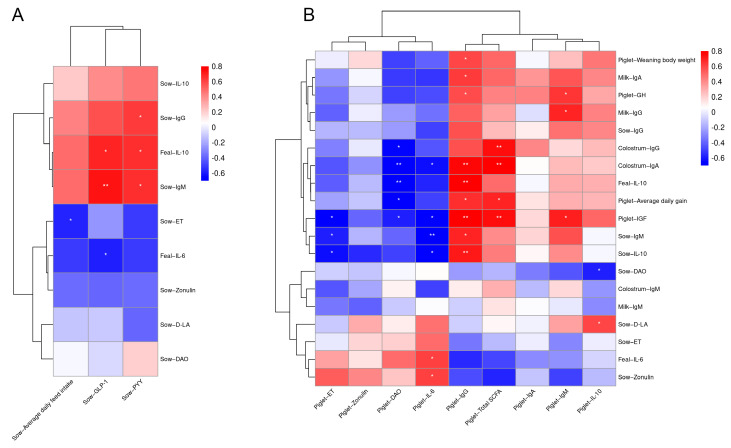
The Pearson correlation analysis of sow feed intake with immunity, inflammation, and intestinal permeability (**A**). Pearson correlation analysis of sow immune level, intestinal permeability, milk immunity, and piglet growth with piglet health status (**B**). The red and blue indicate positive and negative correlations, respectively: * *p* < 0.05 and ** *p* < 0.01.

The correlation analysis of sow feed intake with immunity, inflammation, and intestinal permeability is shown in Figure 7A. The result has shown that sow serum PYY was significantly positively correlated with serum IgG and IgM as well as fecal IL-10. Sow serum GLP-1 levels were significantly positively correlated with serum IgM and fecal IL-10 and significantly negatively correlated with fecal IL-6. Furthermore, the ADFI of sows was significantly negatively correlated with serum ET level. The correlation analysis of sow immune level, intestinal permeability, milk immunity, and piglet growth with piglet health status is shown in Figure 7B. The results showed that the serum ET level of piglets was negatively correlated with the serum IL-10 and IgM of sows and the serum IGF of piglets. The serum DAO levels of piglets were significantly and negatively correlated with the serum IGF and average daily weight gain of piglets, fecal IL-10, and IgA and IgG in colostrum. Piglet serum IL-6 levels were significantly positively correlated with sow zonulin and fecal IL-6, and they were significantly negatively correlated with sow serum IL-10 and IgM, piglet IGF, and colostrum IgA. Piglet serum IgG levels were significantly positively correlated with sow serum IL-10 and IgM, piglet serum IGF and GH, the average daily gain and weaning weight of piglets, fecal IL-10, colostrum IgA, and milk IgA. The content of total short-chain fatty acids in the fecal samples of piglets was positively correlated with serum IGF, average daily gain, and colostrum IgA and IgM. The serum IgM levels of piglets were positively correlated with the serum GH and IGF of piglets and milk IgG. The serum IL-10 of piglets was negatively correlated with the serum DAO of sows and positively correlated with the serum D-LA of sows.

## 4. Discussion

The physiological stages of late gestation and lactation can cause dramatic changes in the metabolism of sows, leading to oxidative stress. This not only affects the health of lactating sows but more importantly reduces the quality of breast milk, ultimately negatively impacting the health and growth of newborn piglets. A healthy diet plays an important role for mothers and babies during gestation and lactation. Therefore, an effective nutrition program is of great significance for sows’ health status and lactation performance in the late gestation and lactation periods [17].

In this study, the addition of CDF to the diet had a tendency to increase weaning litter weight, and it significantly increased weaning BW and average daily gain, which was consistent with [6], who observed that the weaning BW of piglets was increased by supplementing diets with sugar beet pulp in sows. We hypothesize that the improvement in growth performance of lactating piglets was achieved by CDF dietary supplementation through alleviating the oxidative stress of sows and improving milk quality. We then tested indicators of sow health and milk quality to confirm this hypothesis.

Lactation feed intake is very important for the subsequent reproductive performance of sows and the growth and development of suckling piglets [18]. In this study, the lactating feed intake of sows in the CDF group was significantly higher than in the CON group. Indeed, previous studies have shown that dietary fiber supplementation has a positive effect on the feed intake of lactating sows [19,20]. Increased lactation feed intake may be due to dietary fiber supplementation alleviating the systemic inflammatory response of lactating sows by improving their intestinal flora [20]. Another reason may be that supplementing CDF improves insulin sensitivity in sows [21]. Increased lactation feed intake indicates greater maternal energy and nutrient intake, which may improve milk production and quality because at least half of the nitrogen and energy in feed is transferred to milk during peak lactation. Milk production and quality are considered key factors in the growth and development of suckling piglets [22]. Therefore, in this study, the increase in feed intake during lactation may be an important reason for the increase in weaning BW of suckling piglets in the CDF group.

The GH/IGF axis is a master regulator that stimulates the growth of animal cells and somatic cells [23]. Therefore, to investigate whether the effect of maternal CDF on growth performance is related to GH/IGF, we measured serum levels of GH and IGF. The results of this experiment showed that maternal diet supplementation with CDF significantly increased the serum GH and IGF levels of offspring. However, it has been shown that GH achieves promoter growth by regulating IGF-1 synthesis [24]. Therefore, the increased concentrations of GH and IGF may partly explain the improved growth of offspring in sows supplemented with CDF. At present, the underlying mechanism of maternal regulation of the progeny GH/IGF axis has not been clearly explained and needs further study.

Systemic oxidative stress during late pregnancy and lactation is one of the main causes of impaired reproductive performance in sows [25]. GSH-Px and SOD are directly involved in the inactivation of reactive oxygen species as important barriers against oxidative damage in the body, where GSH-Px reduces hydrogen peroxide and SOD plays an important role in the elimination of oxygen radicals [26]. The results showed that CDF diet supplementation increased the activities of GSH-Px and SOD in the serum of sows. Previous studies have also shown that adding dietary fiber (inulin) to a high-fat diet can increase the activities of T-SOD and GSH-Px in the serum of sows and reduce the concentration of MDA [27].

Immunoglobulins are secreted by b-cell activated plasma cells and play an important role in humoral immunity [28]. The main functions of immunoglobulins include the activation of the complement system, which is responsible for inhibiting microbiota attachment and inhibiting bacterial metabolism by blocking enzymes, initiating antibacterial activity, and neutralizing viruses, thereby preventing disease-causing effects [29]. This study showed that feeding CDF-supplemented rations improved IgG and IgM levels in sows. Previous studies have also reported that fiber supplementation can increase immunoglobulin content in pregnant and lactating sows [30]. Notably, IgG in the serum of the sow was the main source of immunity for newborn piglets; specifically, IgG was transferred from the blood to the mammary gland through the FcRn receptor during colostrum formation [31].

Good intestinal barrier function can effectively prevent pathogens, toxins, and antigens from entering the blood circulation through the intestinal mucosa [32]. Therefore, we measured the levels of four serum intestinal permeability-related biomarkers (DAO, D-LA, ET, and zonulin). Although there was no significant difference in serum free of DAO, D-LA, and ET, this study showed that serum zonulin concentration was decreased in sows fed CDF. The levels of zonulin, a gap junction protein, were significantly lower, indicating lower intestinal permeability [33]. Likewise, similar results were obtained in another study in which fiber supplementation improved intestinal barrier function in sows [34]. Improvement in intestinal barrier function may be closely related to increased levels of immunity [20]. Therefore, dietary supplementation with CDF improves the antioxidant capacity of sows and has positive effects on the immune level and intestinal permeability.

Breast milk is the only source of nutrient intake and antioxidant protection for newborn piglets, so colostrum and milk quality play a key role in the growth and development of lactating piglets [25,35]. In this experiment, the supplementation of the diet with CDF increased the activities of SOD and GSH-Px in milk. More importantly, a large number of studies have demonstrated that sow T-SOD, GSH-Px, and MDA can be transmitted to offspring through milk [36,37,38]. In addition, newborn piglets require immunoglobulins absorbed from breast milk for passive humoral immune protection until their own immune system matures [39]. The results of this study showed that the immune level and anti-inflammatory factor IL-10 were significantly higher in the milk and colostrum of the CDF group. High levels of immunoglobulins in breast milk can protect offspring from many pathogens [2]. In addition, it has been shown that increased immunoglobulin content in the colostrum improves intestinal barrier function in piglets [2]. It has been reported that an increase in IL-10 may be transferred from milk to piglet plasma, ultimately affecting the piglet immune system [20]. To further investigate whether maternal CDF supplementation positively affects offspring health status by improving antioxidant capacity and immune levels in milk, we then measured antioxidants, immunoglobulins, inflammatory factors, and intestinal permeability biomarkers in piglet serum. The activity of SOD and GSH-Px and the concentration of IgG and IgM in piglets in the CDF group were significantly higher, and the lipid peroxides MDA and inflammatory factor IL-6 were significantly lower. In addition, the serum intestinal permeability marker DAO was significantly reduced. These results indicate that the offspring of sows supplemented with a CDF diet have better antioxidant capacity and health status and also verify the transmission of antioxidant capacity and immunity between milk and piglet serum. The decrease in intestinal permeability may be closely related to the improvement of immunity and inflammation [40]. It may also be regulated by gut microbes and their metabolites (SCFAs), especially since SCFAs have been shown to have a positive effect on the integrity of the gut barrier [41].

The gastrointestinal microbiota is essential for the metabolism of nutrients, the growth and maturation of immune responses, and the protection of pathogens [42]. As a dynamic ecosystem, gut flora is influenced by many factors, including diet, lifestyle, age, genotype, etc. Diet is particularly important [43]. In this study, the Chao1, Shannon, and Simpson indexes were all significantly improved, indicating that maternal CDF supplementation significantly improved the richness and evenness of intestinal flora. Studies have shown that a more diverse gut microbiome is more stable and healthy than a less diverse gut microbiome, resulting in higher resistance and resilience to chaotic outbreaks of subpopulations and pathogens [44]. We then further investigated the effects of maternal CDF supplementation on microbial composition at the phyla and genus levels. In this study, *Firmicutes* and *Bacteroidetes* were the dominant phyla, and it was found that *Firmicutes* decreased significantly and *bacteroidetes* increased. Studies have reported that the increased ratio of firmicutes to Bacteroidetes may be related to obesity and other diseases, while the decreased ratio of firmicutes to Bacteroidetes may have positive effects on intestinal permeability, metabolic endotoxemia, and inflammation [45]. In addition, this study showed that sows fed CDF-supplemented diets had a higher abundance of *oscillospira* and *lachnospira*. *Oscillospira* belongs to the phylum Firmicutes and is thought to be a fiber-degrading bacterium that can use host glycans as an energy source [46]. In addition, studies have shown that *Oscillospira* has anti-inflammatory properties and is closely related to animal health [47,48]. Meanwhile, *Lachnospira* is considered a potential sign of health. In addition, a significant decrease in the abundance of *Erysipelotrichaceae_UCG-003* was also found in this study. *Erysipelotrichaceae_UCG-003* is believed to have harmful bacterial properties and has been associated with the disruption of bile acid metabolism and intestinal dysfunction [49]. In conclusion, CDF supplementation has positive effects on the intestinal health, immune level, and metabolic homeostasis of sows by improving the intestinal microbiota of sows. Surprisingly, the relative abundance of lactobacillus was significantly reduced in the CDF group, possibly because the intake of the CDF diet led to an increase in the intestinal fibrillary-degrading bacteria.

Short-chain fatty acids, as end products of intestinal microbial fermentation [50], have been shown to have a variety of beneficial effects on immunity and metabolism [51]. This study showed that the fecal content of propionate, butyrate, valerate, isobutyrate, isovalerate, and total SCFAs in the progeny of sows supplemented with CDF diets was significantly increased. Butyric acid, in particular, is considered to be a source of metabolic energy for intestinal cells and has anti-inflammatory properties that help the host maintain healthy intestinal barrier function [52]. Therefore, the increase in the concentration of short-chain fatty acids may be one of the reasons for the decrease in inflammatory factors and the enhancement of immune levels and barrier function of piglets from the CDF-fed sows.

## 5. Conclusions

In summary, maternal CDF supplementation during late gestation and lactation ultimately has significant positive effects on the growth performance, antioxidant capacity, immune levels, cytokines, and intestinal health of piglets by improving sows’ antioxidant capacity and milk quality factors. More importantly, our study provides a potential new option for sow breastfeeding feed.

## Figures and Tables

**Table 1:** Effect of CDF on the performance of lactating sows and suckling piglets ^2^.

Item	CON ^1^	CDF ^1^	SEM ^2^	*p*-Value
ADFI, kg/d	5.39	6.23	0.28	0.007
Backfat thickness, mm				
Initial backfat thickness	18.11	18.83	0.95	0.470
Weaning backfat thickness	16.20	17.18	0.91	0.307
Backfat thickness change	1.92	1.65	0.28	0.368
Litter weight, kg				
Cross-fostering litter weight	20.15	20.10	1.40	0.971
Weaning litter weight	89.91	100.20	6.16	0.111
Litter weight gain	69.75	80.10	5.58	0.079
Mean body weight, kg				
Cross-fostering BW	1.37	0.33	0.08	0.635
Weaning BW	6.11	6.63	0.24	0.042
Average daily gain	0.20	0.22	0.01	0.028
Litter size				
Born alive	14.80	14.67	0.87	0.881
Weaning litter size	11.00	12.45	0.84	0.112
Weaning survival rate, %	0.76	0.86	0.08	0.234

^1^ CON: control; CDF: complex dietary fiber. ^2^ Data are expressed as mean ± SEM (*n* = 12 for each group).

## Data Availability

Data are contained within the article.

## Supplementary Materials

The following supporting information can be downloaded at: https://www.mdpi.com/article/10.3390/antiox13010022/s1, Table S1: Basal diet composition and nutrient levels of late gestation and lactating sows.
