# Supplementary material for: Supplementation with Complex Dietary Fiber during Late Pregnancy and Lactation Can Improve Progeny Growth Performance by Regulating Maternal Antioxidant Status and Milk Quality
Source: Antioxidants (Basel). 2023 Dec 21;13(1):22. doi: 10.3390/antiox13010022 (PMC10812556; doi:10.3390/antiox13010022)
Supplement: Supplementary file 1 [file antioxidants-13-00022-s001.zip › antioxidants-2742304-supplementary.pdf]

Supplementary Materials

Table S1. Basal diet composition and nutrient levels of late gestation and lactating sows.

Items	Late Pregnancy and Lactation
Ingredient amount (%)	
Corn	58.35
Soybean meal	14.50
Puffed soybean	10.00
Wheat bran	6.00
Soybean oil	1.00
Fish meal	2.50
Limestone	1.50
Dicalcium phosphate	0.40
Premix[1]	5.75
Total	100
Nutrition level (calculated value[2])	
Digestible energy (Mcal/kg)	3.37
Crude protein (%)	17.50
Calcium (%)	1.01
Total phosphorus (%)	0.65
L-lysine (%)	0.92
L-methionine (%)	0.26
L-methionine+ L-cysteine, (%)	0.49
L-threonine (%)	0.59
L-tryptophane (%)	0.20

[1] Composition of 1 kg premix: 240–300 KIU VA, 50–125 KIU VD3, 500 IU VE, ≥45 mg VK3, ≥50 mg VB1, ≥150 mg VB2, ≥100 mg VB6, ≥0.5 mg VB12, ≥650 mg niacin, ≥450 mg pantothenic acid, ≥80 mg folic acid, ≥10 mg biotin, 2.4–18 g iron, 0.2–0.62 g copper, 1–2.5 g zinc, 0.5–2 g manganese, 10–50 mg iodine, 5–12.5 mg selenium, 60–220 g sodium chloride, ≥10 mg choline chloride, ≥0.7% L-lysine, ≤10% water. [2] Calculated value: the nutrition levels of basal diet were calculated by an analysis of the crude protein, calcium, total phosphorus and various digestible amino acid levels of corn, soybean meal and wheat bran and DE recommended by NRC.
